# Use of Quantitative Metagenomics Next-Generation Sequencing to Confirm Fever of Unknown Origin and Infectious Disease

**DOI:** 10.3389/fmicb.2022.931058

**Published:** 2022-07-04

**Authors:** Yuxin Dong, Yulei Gao, Yanfen Chai, Songtao Shou

**Affiliations:** Department of Emergency Medicine, Tianjin Medical University General Hospital, Tianjin, China

**Keywords:** quantitative metagenomics next-generation sequencing, fever of unknown origin, infections, pathogen, rare disease

## Abstract

A body temperature >38.3°C that lasts ≥3 weeks and lacks a clear diagnosis after 1 week of standard hospital examination and treatment is called “fever of unknown origin” (FUO). The main causes of FUO are infections, hematological diseases, autoimmune diseases, and other non-infectious inflammatory diseases. In recent years, quantitative metagenomics next-generation sequencing (Q-mNGS) has been used widely to detect pathogenic microorganisms, especially in the contribution of rare or new (e.g., severe acute respiratory syndrome-coronavirus-2) pathogens. This review addresses the undetermined cause of fever and its evaluation by Q-mNGS.

## Introduction

Fever of unknown origin (FUO) can be caused by various diseases. More than 200 causes of FUO have been reported (Horowitz, 2013). In 1961, Petersdorf and Beeson were the first to define FUO as a state of febrile illness for more than 3 weeks, with a body temperature greater than 38.3°C (101°F) on several occasions and an uncertain diagnosis after 1 week of standard hospital examination and treatment (Petersdorf and Beeson, 1961). In 1991, Durak and Street re-defined FUO into four groups: “classic,” “nosocomial,” “neutropenic,” and “human immunodeficiency virus (HIV)-associated.” They proposed three outpatient visits and related investigations as an alternative to “1 week of hospitalization” (Durack and Street, 1991). In 1997, Arnow and Flaherty updated the FUO definition and considered the “minimum diagnostic evaluation to qualify as FUO” to be: comprehensive history-taking; repeated physical examination; complete blood count (including differential and platelet counts); routine blood chemistry (including lactate dehydrogenase, bilirubin, and liver enzymes); urinalysis (including microscopic examination); chest radiograph; erythrocyte sedimentation rate (ESR); antinuclear antibodies; rheumatoid factor; angiotensin-converting enzyme; routine blood cultures (×3) while not receiving antibiotics; cytomegalovirus immunoglobulin-M antibodies or virus detection in blood; heterophile antibody test in children and young adults; tuberculin skin test; computed tomography (CT) of the abdomen or radionuclide scan; HIV antibodies or virus-detection assay; further evaluation of abnormalities detected by the tests stated above (Arnow and Flaherty, 1997). Because of the complicated clinical characteristics and lack of laboratory indicators of a disease, the diagnosis is difficult and contributes to a high cost of hospitalization.

**FIGURE 1:**
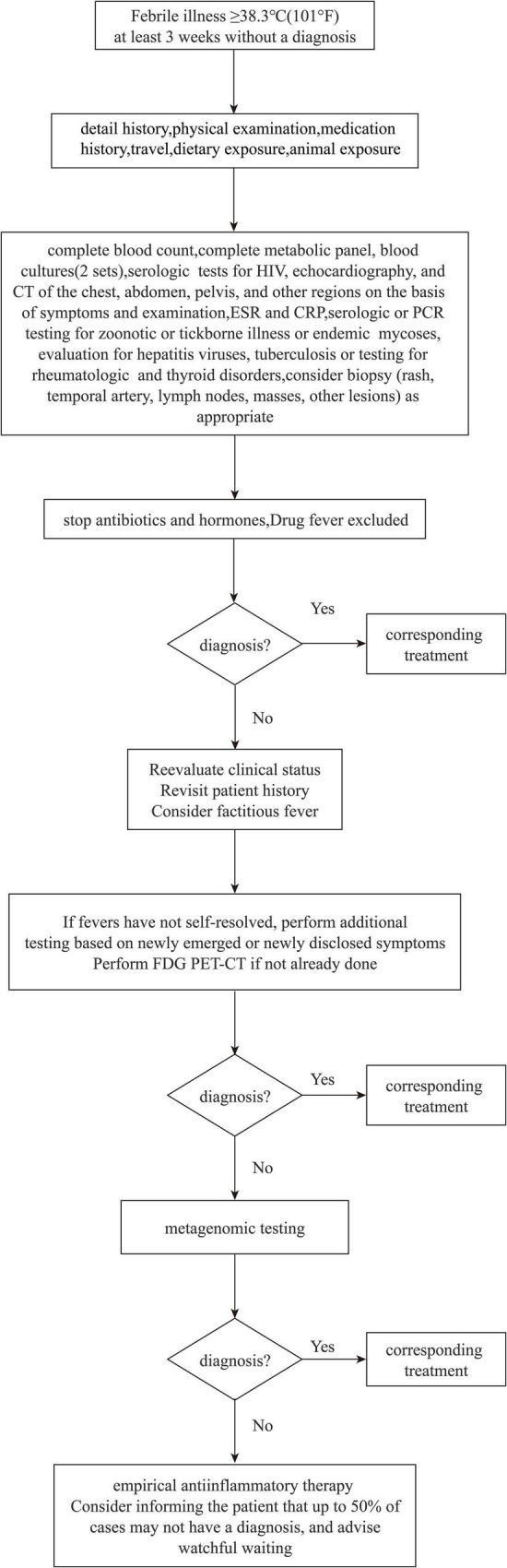
FUO diagnosis and treatment flow chart.

Infections, neoplasms, non-infectious inflammatory diseases, and other conditions are the four primary etiological groups for FUO (Mourad et al., 2003). Obtaining a detailed medical history and undertaking examinations to evaluate the cause of fever are crucial. The standard diagnosis and treatment process of FUO have not yet been proposed, but they should be performed in a specific order when carrying out the examination and diagnosis (Figure 1). Identification of pathogenic bacteria is crucial for targeted anti-infective medication (Haidar and Singh, 2022; Messacar et al., 2017). In patients with prolonged fever, empiric therapy is not recommended because it can mask symptoms, delay the diagnosis, and obstruct decision-making regarding optimal treatment (Unger et al., 2016). Only a few exceptions exist if treatment must be initiated based solely on diagnostic suspicion: antibiotics for culture-negative endocarditis, tuberculostatic agents for active tuberculosis, and glucocorticoids for temporal arteritis with a risk of vision loss (Bryan and Ahuja, 2007). Culture and testing of body fluids are common for a microbial diagnosis, but such cultures and tests are positive in only ∼40% of cases. Also, implementation and interpretation of blood cultures require time, which delays the information obtained by clinicians (Tromp et al., 2012). The sensitivity and specificity of PCR-based detection is based on the genomic sequence of known pathogenic bacteria, provides limited information, and is suboptimal for detection of mixed infections (Reuwer et al., 2019). Medication mistakes or treatment delays may arise due to the limits of clinical testing.

According to two systematic reviews conducted from 1995 to 2004 (Gaeta et al., 2006) and 2005 to 2015 (Fusco et al., 2019), infections are the leading cause of FUO. Screening and diagnostic processes must be developed to detect the pathogens that cause infection-related FUO. Quantitative metagenomics next-generation sequencing (Q-mNGS) is a current method to detect infection-related FUO pathogens. Quantitative metagenomics next-generation sequencing, also known as “high-throughput sequencing” or “massive parallel sequencing,” is a type of technology that allows for the simultaneous and independent sequencing of hundreds to billions of DNA fragments (Morganti et al., 2019). Q-mNGS has many uses in clinical microbiological testing, and provides an unbiased method for pathogen detection. Recent studies have shown that Q-mNGS could be used to diagnose various infectious diseases, including coronavirus disease 2019 (COVID-19) (Ren et al., 2020), pneumonia due to *Chlamydia psittaci* infection (Chen et al., 2020), Ebola virus (EBOV) infection (Li et al., 2019), and talaromycosis (Shi et al., 2021).

### Revolution in DNA-Sequencing: From Sanger Sequencing to Quantitative Metagenomics Next-Generation Sequencing

The “first generation” of gene-sequencing technology was born with the advent of the chain-termination method described by Sanger and Coulson (1975) and the chain-degradation method described by Maxam and Gilbert (1977). Gilbert and Sanger built the first sequencer in 1977 and used it to sequence the first full-length genome, phage X174, with 5,375 bases (Maxam and Gilbert, 1977; Aoyama et al., 1981). First-generation sequencing can produce a sequence of 700–1,000 bases at a time, so it cannot keep up with the pressing need for biological gene sequences.

Following a revolution in traditional sequencing technology, second-generation sequencing technology, known as “next-generation sequencing” (NGS), can be employed to obtain the sequences of hundreds of thousands to millions of nucleic-acid molecules in a single run. With NGS introduction, the transcriptome and genome of a species can be investigated in great detail. Jonathan Rothberg developed the biotechnology company 454 Life Sciences (Branford, CT, United States) in 2005 (Margulies et al., 2005). Other technologies, such as sequencing by oligonucleotide ligation and detection (SOLiD) (Applied Biosystems, Foster City, CA, United States) and Solexa (Illumina, San Diego, CA, United States), emerged subsequently. A total of 454 Life Sciences was acquired by Roche (Basel, Switzerland) in 2007.

The basic principles of this technology are that the DNA fragment does not need to be fluorescently labeled, there is no need for electrophoresis, and the sequence is changed by synthesis. A pyrophosphate group is removed when a base is added to the sequence, so pyrosequencing is also known as the detection of pyrophosphate bases (Nyrén et al., 1993; Ronaghi et al., 1998). Sequencing by SOLiD technology is based on ligase sequencing (Pérez-Enciso and Ferretti, 2010). Solexa technology (which is also used for sequencing-by-synthesis) was developed first by Illumina and is now used by the second-generation sequencer developed by Illumina (Pérez-Enciso and Ferretti, 2010; Strub et al., 2011).

Metagenomics (also known as “microbial environmental genomics”) creates a metagenomic “library” by extracting the DNA or RNA of all microorganisms from environmental samples directly and studying them using genomics research strategies. Metagenomics based on NGS has become the focus of clinical research since the development of gene-sequencing technology.

Q-mNGS is a method for analyzing the genetic material of microbes and hosts from patient samples to diagnose infectious diseases. Q-mNGS has become the focus of clinical research due to the rapid advancement of gene-sequencing technology.

**FIGURE 2:**
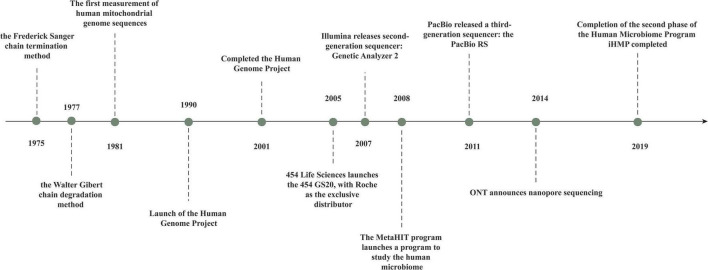
The history of gene sequencingtechnology.

Third-generation sequencing technology includes the Pacific Bioscience (Levene et al., 2003) and Oxford Nanopore (Eisenstein, 2012) platforms, which are single-molecule technologies. Single-molecule sequencing (which does not require PCR amplification and which can, theoretically, determine nucleic-acid sequences of any length) is most notable when compared with first-generation and second-generation sequencing technologies (Figure 2).

### Quantitative Metagenomics Next-Generation Sequencing in Fever of Unknown Origin or Infectious Diseases

The diagnostic value of NGS has been investigated in retrospective studies for patients suffering from fever (Table 1). The effectiveness of detection of NGS is higher than that of traditional methods. Fu et al. (2021) undertook a retrospective study on 175 patients with FUO to compare Q-mNGS with culture and traditional methods, including smears, serological tests, and amplification of nucleic acids (traditional PCR, Xpert MTB/RIF, and Xpert MTB/RIF Ultra). In comparison with culture and conventional methods, the authors concluded that Q-mNGS of blood might increase the overall rate of detection of novel organisms by 22.9 and 19.79%, and enhance the diagnostic rate by 38.0 and 32.0%, respectively. Zou et al. (2022) evaluated 12 patients with tuberculosis following renal transplantation, and Q-mNGS was helpful in 67% of cases.

**TABLE 1:** List of sequencing associated with fever or infectious disease’ study.

References	Research type	Disease type	Method	Conclusion
Li et al. (2019)	Retrospective	70 patients of suspected Ebola hemorrhagic fever	mNGS vs. qRT-PCR_US_	These results demonstrate the utility of mNGS in broad-based pathogen detection and outbreak surveillance.
Fu et al. (2021)	Retrospective	175 patients of FUO	mNGS vs. culture or traditional methods [smears, serological tests, nucleic acid amplification testing (NAAT)]	mNGS had significantly higher diagnostic efficacy in the FUO than culture or other traditional methods.
Zou et al. (2022)	Retrospective	12 patients of mycobacterium tuberculosis infection	Interferon-gamma release assay and NGS vs. the traditional PPD test and M. tuberculosis detection	The interferon-gamma release assay and NGS are relatively new detection methods with high sensitivity and specificity and can help with early TB diagnosis.
Benamu et al. (2021)	Prospective	55 patients with febrile neutropenia (FN)	The Karius microbial cell-free DNA (mcfDNA) sequencing Test (KT) vs. blood culture (BC) and standard microbiological testing (SMT)	The use of KT in the diagnosis and treatment of FN shows promise.
Liu et al. (2020)	Retrospective	17 patients of underwent lung transplantation	NGS vs. the bacteria culture method	NGS showed more sensitivity than bacterial culture for the detection of bacteria.
Reyes et al. (2021)	Retrospective	38 patients of febrile illness	mNGS vs. conventional viral pathogen detection methods (such as PCR)	In international travelers with febrile syndrome, viral metagenomics has the potential to help identify viral pathogens and co-infections in a single step.
Xiao et al. (2020)	Retrospective	8 patients of COVID-19	Meta sequencing vs. multiplex PCR amplification (amplicon) and hybrid capture (capture)	Meta-sequencing can be prioritized if other genetic materials are to be studied, such as target viruses that have become highly diversified through recombinational events, or if the viral load within the RNA sample is high.
Jerome et al. (2019)	Retrospective	40 patients of fever after traveling	mNGS analysis vs. standard of care diagnostics	MNGS has the potential to improve infectious disease diagnostic yield and detect multiple pathogens in a single sample.
Williams et al. (2018)	Retrospective	12 plasma specimens from patients with unexplained febrile illness	Unbiased sequencing vs. VirCapSeq-VERT (a positive selection system).	The utility of high-throughput sequencing strategies in outbreak investigations
Horiba et al. (2021)	Retrospective	112 patients of pediatric febrile neutropenia	NGS vs. blood cultures	NGS technique has great potential for detecting causative pathogens in patients with FN and may be effective for detecting pathogens in minute quantities of microbiota.

Benamu et al. (2021) evaluated 55 patients with febrile neutropenia to compare the results of blood culture and standard microbiological testing within 24 h of fever onset and every 2–3 days. The Karius microbial cell-free DNA sequencing test (KT) sensitivity and specificity were 85% (41/48) and 100% (14/14), respectively. The calculated time-to-the-diagnosis was, in general, shorter with KT (87%). Adjudicators determined real-time KT results have allowed early optimization of antimicrobial agents in 47% of patients. Liu et al. (2020) retrospectively evaluated 17 patients who received a lung transplant. The proportion of bacteria detected in the lungs of donors was 52.9 and 35.3% by NGS and bacterial culture, respectively. NGS was more sensitive for bacterial detection than the classic bacterial culture. Reyes and colleagues Xiao undertook a retrospective study on 38 patients. In eight of the 38 patients (21%), all viral pathogens detected by 42 conventional assays were also detected by Q-mNGS, and Q-mNGS resulted in additional pathogenic findings in two patients (5%).

NGS provides more information than conventional diagnostic tests. Xiao et al. (2020) were the first to systematically investigate inter- and intra-individual variations in severe acute respiratory syndrome-coronavirus-2 (SARS-CoV-2) using amplicon- and capture-based whole-genome sequencing; it was also the first comparative study using multiple approaches. The study illustrated that ultra-high-throughput metatranscriptomic (meta) sequencing uncovered rich genetic information in clinical samples besides SARS-CoV-2, and provided references for clinical diagnostics and therapeutics. In June 2020, the US Food and Drug Administration granted Emergency Use Authorization for a Q-mNGS test for COVID-19 manufactured by Illumina, the first such authorization for a NGS diagnostic News in Brief (2020). Li et al. (2019) demonstrated that Q-mNGS of field-collected samples could be used to recover nine genomes from the EBOV outbreak in Boende (Democratic Republic of Congo) in 2014 (>50% coverage), detect the EBOV with a high sequencing depth of 17.3 ± 4.7 *SD* million reads with comparable sensitivity to PCR, and identify co-infections from well-recognized (*Plasmodium falciparum*) and novel/uncommon (e.g., Orungo virus) pathogens. Jerome et al. (2019) prospectively included 40 returning travelers presenting with fever (≥38°C) whose plasma samples were sequenced: 11 of 40 patients were diagnosed with a viral infection. Five viral infections were detected by Q-mNGS that were also revealed in standard-of-care diagnostics, but two patients infected with the Chikungunya virus and one patient with the mumps virus were also diagnosed by Q-mNGS only. Williams et al. (2018) investigated the plasma virome from cases of unexplained febrile illness in Tanzania from 2013 to 2014 by sequencing methods. The latter could aid detection of viral coinfections, such as the nearly complete genomes of dengue virus-2 and human pestivirus. Horiba et al. (2021) evaluated 87 patients with febrile neutropenia. Putative pathogens were detected by Q-mNGS in 17.2% of patients, but all had negative blood cultures. Pathogenic detection methods (e.g., PCR) require clinicians to first suspect a specific bacterial infection before carrying out the corresponding detection. However, NGS technology can be employed to detect pathogenic bacteria in patient samples with high sensitivity, thereby providing recommendations for clinical treatment.

Often, NGS has been undertaken without using a structured diagnostic protocol, and at different stages of FUO. Zhu et al. (2021) found that use of Q-mNGS for blood as the first-line investigation could increase the diagnosis rate of FUO by 10.9% compared with that using culture, and that using Q-mNGS as the second-line investigation could improve the diagnosis rate of concurrent infection by 66.7 and 12.5% for non-bloodstream infection.

Ultimately, the cost of FUO assessment can be reduced by Q-mNGS application because the diagnosis will be achieved early because unnecessary and costly diagnostic tests will not be carried out. Chai et al. (2018) investigated the cost–benefit relationship of Q-mNGS in FUO in which a cause could not be found despite appropriate investigations. A decision tree was created to describe systematically the costs and benefits associated with NGS introduction. Each diagnostic pathway was made until a first- or second-line investigation was positive. NGS was introduced into the pathway as a supplement to first- or second-line investigations. Chai and colleagues reported NGS use as the first-line investigation assuming a probability of detecting the cause of cost-effectiveness in all cases of ≥60% using unit costs of diagnostic tests and procedures in Singapore dollars in 2016. In that analysis, using a rational set of rates for a second-line investigation, the total expected cost of using NGS as a second-line investigation was greater than that using it as a first-line investigation. Although that analysis excluded the costs associated with hospitalization duration, the faster and more definitive answers provided by NGS may enable additional cost savings.

## Conclusion

Q-mNGS is a sensitive diagnostic method for FUO evaluation. It could become a routine procedure in the diagnostic workup of FUO. Q-mNGS appears to be cost-effective in FUO because it avoids unnecessary investigations and reduces the duration of hospitalization.

## Author Contributions

Y-LG and S-TS conceived this study. Y-XD and Y-LG collected clinical data and were responsible for patient care, and drafted the manuscript. Y-LG, S-TS, and Y-FC revised the manuscript critically. All authors have revised the final version of the manuscript and approved it for publication.

## Conflict of Interest

The authors declare that the research was conducted in the absence of any commercial or financial relationships that could be construed as a potential conflict of interest.

## Publisher’s Note

All claims expressed in this article are solely those of the authors and do not necessarily represent those of their affiliated organizations, or those of the publisher, the editors and the reviewers. Any product that may be evaluated in this article, or claim that may be made by its manufacturer, is not guaranteed or endorsed by the publisher.
